# Cryo-thawed embryo transfer: natural versus artificial cycle. A non-inferiority trial.(ANTARCTICA trial)

**DOI:** 10.1186/1472-6874-12-27

**Published:** 2012-09-05

**Authors:** Eva R Groenewoud, Nick S Macklon, Ben J Cohlen

**Affiliations:** 1 UMCG, Groningen, The Netherlands; 2 Complete Fertility Centre, Department of Obstetrics and Gynaecology, Division of Human Development and Health, University of Southampton, Southampton, UK; 3 Fertility Centre Isala Clinics, Zwolle, The Netherlands

## Abstract

**Background:**

Frozen thawed embryo transfer (FET) is a cost- effective adjunct to IVF or IVF-ICSI treatment. In order to optimize treatment outcome, FET should be carried out during a period of optimal endometrial receptivity. To optimize implantation several methods for endometrium preparation have been proposed. In natural cycle FET (NC-FET), the endometrium develops under endogenous hormonal stimulation. The development of the dominant follicle and endometrium is monitored by ultrasound and FET is timed after triggering ovulation induction or determination of the spontaneous LH surge. In an artificial cycle FET (AC-FET) estrogens and progesterone are administered to prepare the endometrium for implantation. While the currently available data show no significant difference in pregnancy rates between these methods, well designed randomized controlled trials are lacking. Moreover there is little literature on difference in cancellation rates, cost-efficiency and adverse events.

**Methods and design:**

In this randomized, multi-centre, non-inferiority trial we aim to test the hypothesis that there is no significant difference in live birth rates between patients undergoing NC-FET versus AC-FET. The primary outcome will be live birth rate per embryo transfer procedure. Secondary outcomes will be ongoing and clinical pregnancy rate, cancellation rate, (serious) adverse events and cost-efficiency. Based on a live birth rate of 20% and a minimal clinical important difference of 7,5% (one-sided alpha 2,5%, beta 20%) a total of 1150 patients will be needed. Analyzes will be performed using both per protocol as well as intention to treat analyses.

**Discussion:**

This prospective, randomized, non –inferiority trial aims to address the hypothesis that there is no significant difference in live birth rates between patients undergoing NC-FET versus patients undergoing AC-FET. Moreover it addresses cost-efficiency as well as the perceived burden of both treatments.

**Trial register:**

Netherlands trial register (NTR): 1586

## Background

In Vitro Fertilization (IVF) or Intra Cytoplasmatic Sperm Injection (ICSI) treatment cycles often produce more embryos than can be transferred during the fresh treatment cycle. Moreover, in some patients embryo transfer is postponed for medical reasons (e.g. ovarian hyperstimulation syndrome). Cryopreservation of these embryos provides both physicians and patients a safe, successful and presumably cost-efficient option [1-3]. Recent development and implementation of single embryo transfer strategies in IVF and IVF -ICSI programs has increased the importance of successful frozen thawed embryo transfer (FET) programs. Critical for a successful FET program is synchronization between the endometrial development and the embryo [4-7]. To achieve this, FET requires extensive preparation, timing and planning. In recent years, several methods for endometrium preparation have been developed.

In natural cycle FET (NC-FET) planning of embryo thawing and transfer requires the identification of a period of optimal receptivity [4-7]. This putative ‘window of implantation’ starts shortly after ovulation. If an embryo is transferred within this window, the chances of conceiving are greater [4-6]. Planning NC-FET can either be done based on recognition of the LH surge that precedes ovulation (using serum or urine LH monitoring) or by triggering ovulation (sometimes referred to as modified NC-FET). Using modified NC-FET, the development of the dominant follicle is closely monitored by regular ultrasonic evaluation. On reaching a diameter of 16-20 mm human chorionic gonadotrophin (hCG) is administered and ovulation takes place approximately 36 h later. Embryo thawing and transferring can be planned accordingly. Despite ultrasonic monitoring, spontaneous ovulations do occur. In such an event the start of the window of implantation cannot be estimated accurately. Since identification of the onset of the window of implantation is mandatory for further timing of FET, cycles with spontaneous ovulation are usually cancelled. To minimize cancellation, patients are required to visit their clinic several times which is time consuming and expensive. In NC-FET cycles, 5-6% of all patients have insufficient development of the dominant follicle and/or endometrium thickness and treatment has to be cancelled [8]. However, a clear advantage of NC-FET is the fact that it does not require patients to take medication for several weeks. In summary, NC-FET has the advantage of not requiring medication but this advantage is balanced against the need for frequent ultrasonic evaluation of the dominant follicle, the risk of unexpected ovulation and the risk of insufficient development of the endometrium and/or dominant follicle. Due to these factors NC-FET is more difficult to plan.

Because of the above mentioned disadvantages an artificial FET (AC-FET) has been developed. This treatment, which was originally developed for patients undergoing oocyte donation, was also found to be successful for patients undergoing FET treatment [9]. During AC-FET patients start with daily estrogens which are supplemented with progesterone when the endometrial thickness is considered sufficient. Patients have to take these drugs for several weeks. The main advantage of this treatment is that it requires little ultrasonic monitoring and therefore is more easily scheduled placing less burden on both patients and doctors agenda’s. More over, planning thawing and transfer is flexible and can be performed based on convenience. Some also claim that supplementing estrogen reduces cancellation rates due to insufficient endometrium thickness compared to NC-FET. The main disadvantages of AC-FET are possible side-effects and higher risk of thrombo-embolic events [10,11].

In recent years several, retrospective, studies comparing live birth rates in both NC-FET and AC-FET have been published. Both Morovoz et al. and Chang et al. concluded that NC-FET results in higher pregnancy rates [12,13]. However, in a retrospective analysis of 1677 FET cycles, Givens et al. observed no difference in pregnancy rates between NC-FET and AC-FET. Pregnancy rates did not differ significantly between both groups [14]. These results were consistent with those of others [15-17]. A recent Cochrane review on treatment regimes in FET concluded that current evidence does not demonstrate a significant difference in pregnancy rates between these methods of endometrial preparation. However, the authors highlighted the need for further a well designed, adequately powered RCT [18].

To the best of our knowledge there is no literature on available discerning which regimen patients prefer, which has fewer side effects, or which is the most cost effective [19]. If live birth rates indeed are equal in NC-FET and AC-FET the perceived burden of both treatments, convenience and cost-efficiency might be important factors in choosing one of both options.

## Methods and design

### Study objective

When it comes to planning FET treatment several questions remain. Although retrospective trials found no significant difference in pregnancy rates there is little literature on other aspects such as side-effects and cost-efficiency. This prospective, randomized, controlled trial aims to address these questions. All patients participating in the ANTARCTICA trial will also be invited to fill in questionnaires regarding the perceived burden of both treatments. The protocol for this study (Penguin-study) will be discussed separately.

### Hypothesis

The hypothesis to be tested is that there is no significant difference in live birth rates between NC-FET and AC-FET, but that NC-FET is more cost effective. Since the expected low incidence of serious adverse events we expect to find no significant difference based on a type 1 error.

### Study design

**Figure 1:**
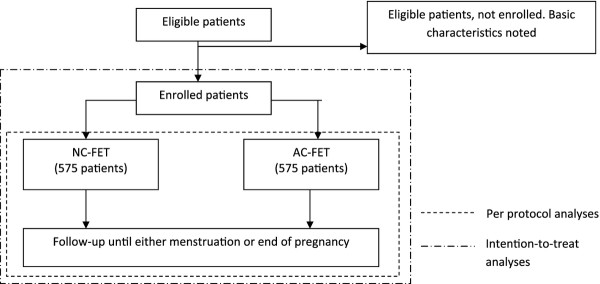
Flowchart eligible patients.

The ANTARCTICA trial is a multi-center, randomized controlled trial, powered to demonstrate non-inferiority of NC-FET in terms of the primary study end point. A recent systemic review of current literature identified no other RCTs addressing this subject. Several retrospective studies have shown discrepant findings but pooling of the data indicated no difference in pregnancy rates between NC-FET and AC-FET. Since our hypothesis is that significant difference in the primary study endpoint will not be observed, but a difference in cost-efficacy in favour of the NC-FET will be demonstrated, a non-inferiority design is appropriate. The research team is blinded for the result of the randomization (Figure 1).

### Study population and recruitment

Patients participating in the ANTARCTICA trial are between the age of 18 and 40 and are undergoing FET after their 1^st^, 2^nd^ or 3^rd^ IVF or IVF-ICSI cycle for various indications. They must all have ovulatory cycles between 26 and 35 days and be willing to give written informed consent to participate in the study.

Those patients who have a known contra-indication or allergy for oral estradiol or vaginally administered micronized progesterone are not eligible. Patients with a uterine anomaly are also excluded from participation. Oocyte donation for any other than a genetic indication is also an exclusion criterion.

Eligible patients are informed about the ANTARCTICA trial during intake by their physician prior to FET cycle. A minimum reflection period of 5 days will be offered. Eligible patients who wish to participate will be randomized after providing written consent. If an eligible patient declines participation, some basic characteristics will be obtained to identify any significant selection bias.

### Primary and secondary endpoints

The primary endpoint is live birth rate per FET. Secondary endpoints are cancellation rate, clinical pregnancy rate, ongoing pregnancy rate (all per FET), endometrium thickness during transfer and (serious) adverse events. More over cost-efficiency calculations will be performed.

### Participating hospitals

Both secondary and tertiary fertility clinics performing IVF and IVF-ICSI treatment are invited to participate in the ANTARCTICA trial. At present 15 Dutch fertility clinics are actively enrolling patients. Two further clinics will join, pending approval by their local ethics committee.

### Randomization

Randomization is performed using a web based randomization program, based on restricted randomization with allocation clusters of alternating sizes. Allocation is based on a 1:1 assignment. Stratification for the initial treatment (IVF vs IVF-ICSI) is performed. Since live birth rates might differ between clinics, each clinic has its own blinded allocation list.

### Data collection

Data collection is performed using a web-based case report form (electronic CRF or eCRF). Basic data on patient’s general history and specific fertility history are obtained from the out-patient clinic chart. Characteristics of the initial treatment as well as characteristics of the FET treatment are also noted.

For the cost-efficiency study patients are invited to answer a web-based questionnaire after completing treatment.

### Interventions

#### NC-FET (intervention 1)

Patients allocated to intervention 1 will undergo their FET in a natural cycle. Starting on day 10, 11 or 12 of their cycle regular ultrasonic evaluation of the endometrium thickness and mean diameter of the dominant follicle is performed. When the endometrium is 6 mm or more and the diameter of the dominant follicle is 16-20 mm a blood sample is taken (for blinded analyses of progesterone and LH levels) and ovulation is induced using hCG injection (pregnyl 5000 IE, MSD USA or ovitrelle 250 μgram, Serono Benelux bv, Germany). Thawing and transferring is performed subsequently according to local protocols. A maximum of two embryos will be transferred.

**Figure 2:**
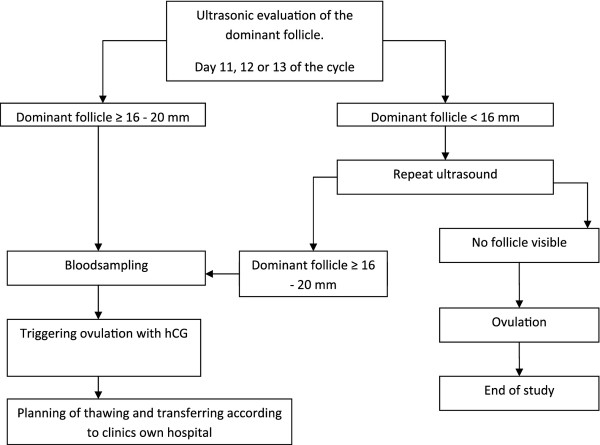
Flowchart natural cycle.

If during ultrasonic evaluation no follicle is visible ovulation is deemed to have occurred. In this event no thawing or transferring will take place. This cycle is regarded a drop-out. Further treatment can be conducted according to local protocols (Figure 2).

#### AC-FET (intervention 2)

If patients are allocated to intervention 2, an artificial cycle FET will be performed. From day one of the cycle patients commence oral estradiol (progynova, Bayer, Germany) 2 mg three times daily. After 11, 12 or 13 days an ultrasound is performed. If no leading follicle is present and the endometrial thickness is ≥ 8 mm, micronized progesterone (utrogestan Besins International, Belgium) is added to the regime and thawing and transferring is commenced 4 or 5 days later according to the stage of cryopreservation [20].

If the endometrial thickness is less than 8 mm, the progynova dose is raised to 2 mg 4 times daily for 7 days. After a week the endometrium is checked once again. When the endometrium thickness is >8 mm and no dominant follicle (≥ 14 mm) is present, utrogestan can be added and thawing and transferring is performed according to local protocols. A maximum of two embryos will be transferred.

**Figure 3:**
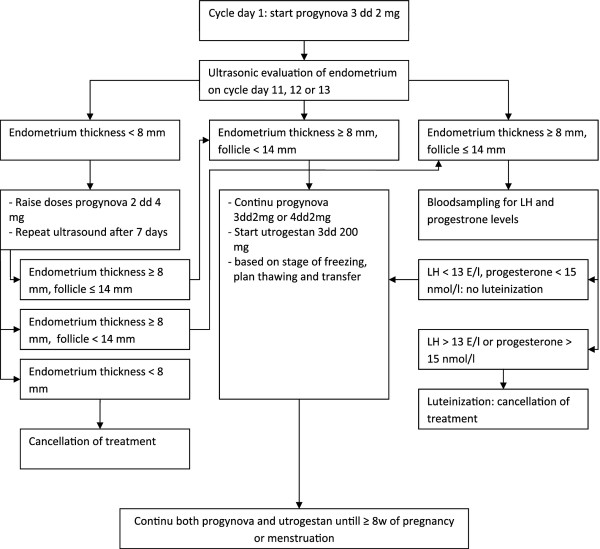
Flowchart artificial cycle.

If a follicle is visible during ultrasound, serum luteinizing hormone (LH) and progesterone levels are determined. If these are raised, (serum LH ≥ 13 E/l or progesterone ≥ 15 nmol/l) luteinization of the follicle is considered to have taken place and because of the associated diminished pregnancy rates, thawing and transferring will not be performed. If serum levels are below the above mentioned levels thawing and transferring can be performed according to local protocol (Figure 3).

Embryo quality is recorded according to study criteria. These criteria can be used beside clinics’ own criteria. The criteria for embryo quality are based on the consensus between the largest participating centers.

### Statistical analysis

#### Sample size and power considerations

Live birth rates in FET treatment in the Netherlands are, on average, 20% per cycle. The minimal clinical important difference (MCID) could not be based on literature concerning FET treatment alone. Based on other studies within fertility care a consensus of a MCID of 7,5% was adapted. This clinical important difference compares to a one sided hypothesis test with a 0.025% significance level with a null hypothesis of AC-FET being inferior to NC-FET. Using a two-sided alpha of 5% and 80% discriminating capacity we calculated that 1150 patients have to be enrolled in this study. All patients will participate with one cycle only.

#### Data analysis

Statistical analyses will be performed using a per protocol analyses. The primary endpoint in this study is live birth rates in both treatments. Since intention-to-treat analyses is more adequate for implementing the results of this study in daily fertility practice such an analyses will be performed also besides a per-protocol analysis. Using logistic regression analysis confounding factors will be analyzed. These factors include age during initial treatment, duration of the subfertility, number and quality of the embryos transferred and outcome of initial treatment. Selection of these criteria is based on current opinion in literature [20-24]. A comparison of baseline characteristics of enrolled patients with the baseline characteristics of patients who refused enrollment will be performed to exclude selection bias.

Secondary endpoints are clinical and ongoing pregnancy rates, cancellation rate, endometrium thickness and (serious) adverse events. Most secondary endpoints will be analyzed using a Fishers’ exact test; endometrium thickness will be analyzed using a student *t*-test. Cost-efficiency of both treatments will be calculated on the ratio between differences in cost and live birth rates.

### Ethics

This study is designed using the guidelines for good clinical practice as well as the decleration of Helsinki. Approval was obtained from both national (CCMO) and Medical Ethics Committee of the Isala Clinics in Zwolle. For each participating hospital approval of the local Medical Ethics Committee was requested. According the GCP guidelines written informed consent prior to randomization will be mandatory.

## Discussion

With this study we aim to clarify whether NC-FET and AC-FET do not differ significantly in live birth rates. Moreover we hope to provide some answers regarding secondary endpoints and cost-efficiency.

The use of hCG in planning NC-FET remains a subject of debate. In recent years both randomized prospective studies as well as retrospective studies have been publicized with conflicting results. In this study NC-FET based on ovulation induction was chosen after careful considerations. There are several issues when using LH determination for timing FET. (Groenewoud et al., Reprod Biomed Online. 2012 Feb;24 (2):191–6) Also there is no information on whether pregnancy rates could be improved if we would adjust planning of thawing and transfer according to the presence of LH surge. To the best of our knowledge, no such studies have been conducted in patients undergoing ultrasound monitored unstimulated cycle FET.

Due to the lack of literature in FET treatment some of our decisions regarding study design and statistical analyses (e.g. MCID) had to be made based on IVF literature in general. With 15 clinics participating the actual live birth rate might differ from clinic to clinic. Choosing both an overall analyses as well as analyses per clinic the authors hope to gain insight in this matter. Moreover choosing a MCID of 7,5% results in a relative high number of patients needed for sufficient statistical power. In designing this study, careful consideration was therefore required of the feasibility of meeting recruitment targets, and what steps could be taken to limit obstacles to recruitment. With these considerations, and the participation of 15 clinics, we consider this study to be achievable within the planned time frame of 4 years.

## Competing interests

The authors declare to have no financial or non financial conflicts of interest. An education grand was awarded by MSD, the Netherlands. MSD had by no means any part in the realization of this study.

## Authors' contributions

ERG and BJC developed and initiated this study. ERG drafted this manuscript and is responsible for the logistics of the trial. All authors read and approved the final manuscript.

## Pre-publication history

The pre-publication history for this paper can be accessed here:

http://www.biomedcentral.com/1472-6874/12/27/prepub
